# Supplementary material for: Synthesis and Bioevaluation of New Stable Derivatives of Chrysin-8-C-Glucoside That Modulate the Antioxidant Keap1/Nrf2/HO-1 Pathway in Human Macrophages
Source: Pharmaceuticals (Basel). 2024 Oct 17;17(10):1388. doi: 10.3390/ph17101388 (PMC11510274; doi:10.3390/ph17101388)
Supplement: Supplementary file 1 [file pharmaceuticals-17-01388-s001.zip › pharmaceuticals-3250969-supplementary.pptx]

## Slide 1
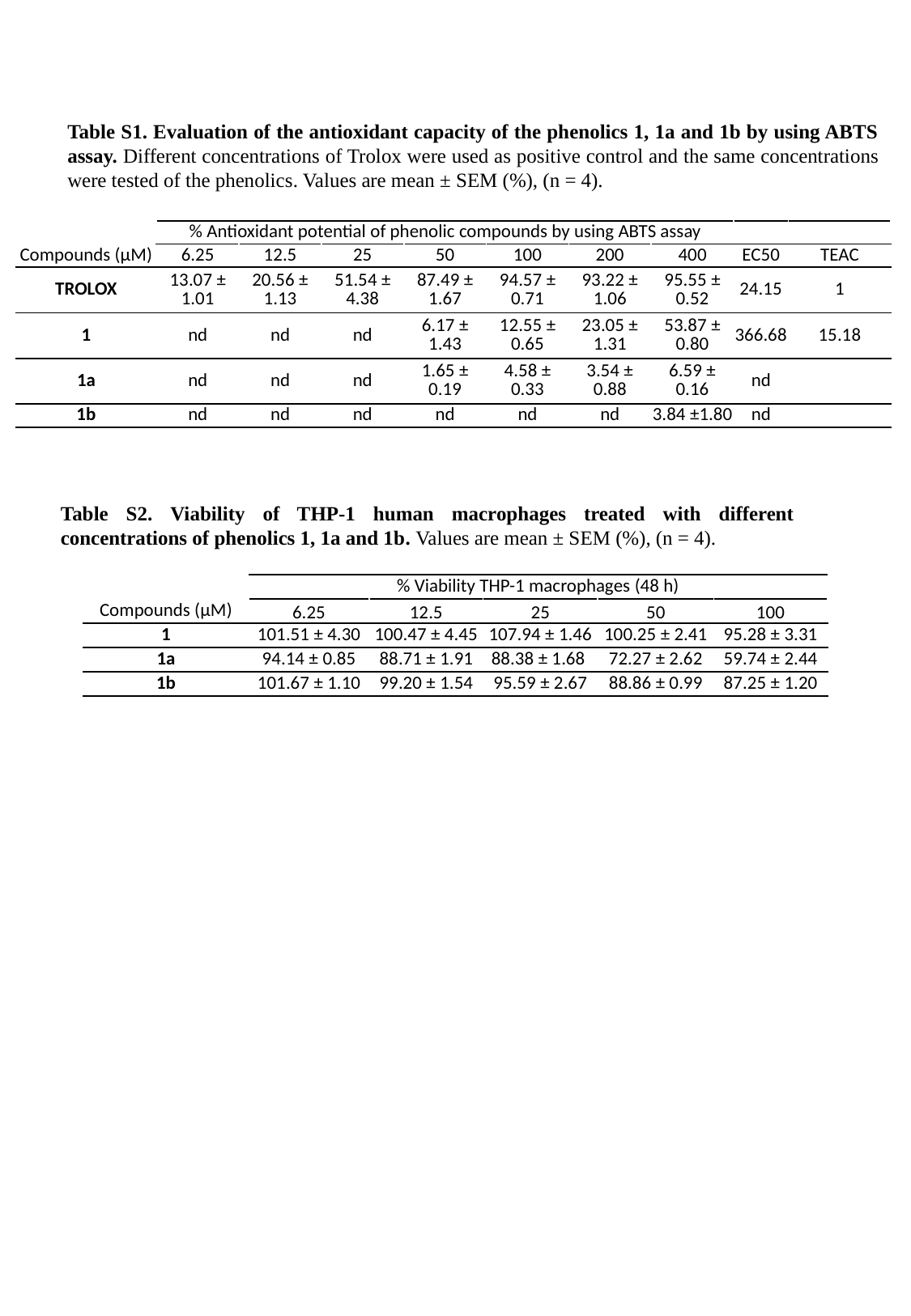

Table S1. Evaluation of the antioxidant capacity of the phenolics 1, 1a and 1b by using ABTS assay. Different concentrations of Trolox were used as positive control and the same concentrations were tested of the phenolics. Values are mean ± SEM (%), (n = 4).
| | % Antioxidant potential of phenolic compounds by using ABTS assay | | | | | | | | |
| --- | --- | --- | --- | --- | --- | --- | --- | --- | --- |
| Compounds (µM) | 6.25 | 12.5 | 25 | 50 | 100 | 200 | 400 | EC50 | TEAC |
| TROLOX | 13.07 ± 1.01 | 20.56 ± 1.13 | 51.54 ± 4.38 | 87.49 ± 1.67 | 94.57 ± 0.71 | 93.22 ± 1.06 | 95.55 ± 0.52 | 24.15 | 1 |
| 1 | nd | nd | nd | 6.17 ± 1.43 | 12.55 ± 0.65 | 23.05 ± 1.31 | 53.87 ± 0.80 | 366.68 | 15.18 |
| 1a | nd | nd | nd | 1.65 ± 0.19 | 4.58 ± 0.33 | 3.54 ± 0.88 | 6.59 ± 0.16 | nd | |
| 1b | nd | nd | nd | nd | nd | nd | 3.84 ±1.80 | nd | |
Table S2. Viability of THP-1 human macrophages treated with different concentrations of phenolics 1, 1a and 1b. Values are mean ± SEM (%), (n = 4).
| | % Viability THP-1 macrophages (48 h) | | | | |
| --- | --- | --- | --- | --- | --- |
| Compounds (µM) | 6.25 | 12.5 | 25 | 50 | 100 |
| 1 | 101.51 ± 4.30 | 100.47 ± 4.45 | 107.94 ± 1.46 | 100.25 ± 2.41 | 95.28 ± 3.31 |
| 1a | 94.14 ± 0.85 | 88.71 ± 1.91 | 88.38 ± 1.68 | 72.27 ± 2.62 | 59.74 ± 2.44 |
| 1b | 101.67 ± 1.10 | 99.20 ± 1.54 | 95.59 ± 2.67 | 88.86 ± 0.99 | 87.25 ± 1.20 |

## Slide 2
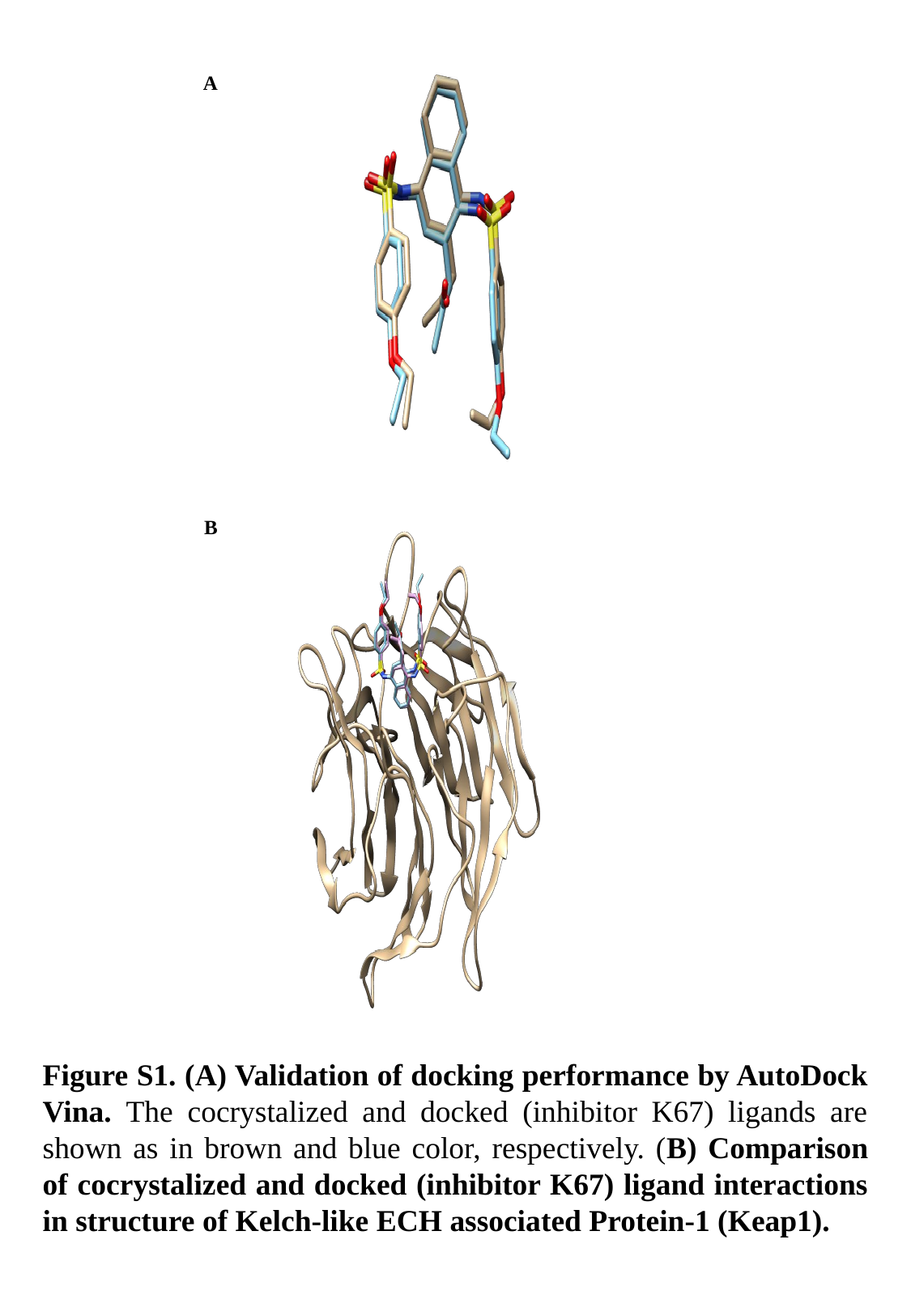

A
B
Figure S1. (A) Validation of docking performance by AutoDock Vina. The cocrystalized and docked (inhibitor K67) ligands are shown as in brown and blue color, respectively. (B) Comparison of cocrystalized and docked (inhibitor K67) ligand interactions in structure of Kelch-like ECH associated Protein-1 (Keap1).

## Slide 3
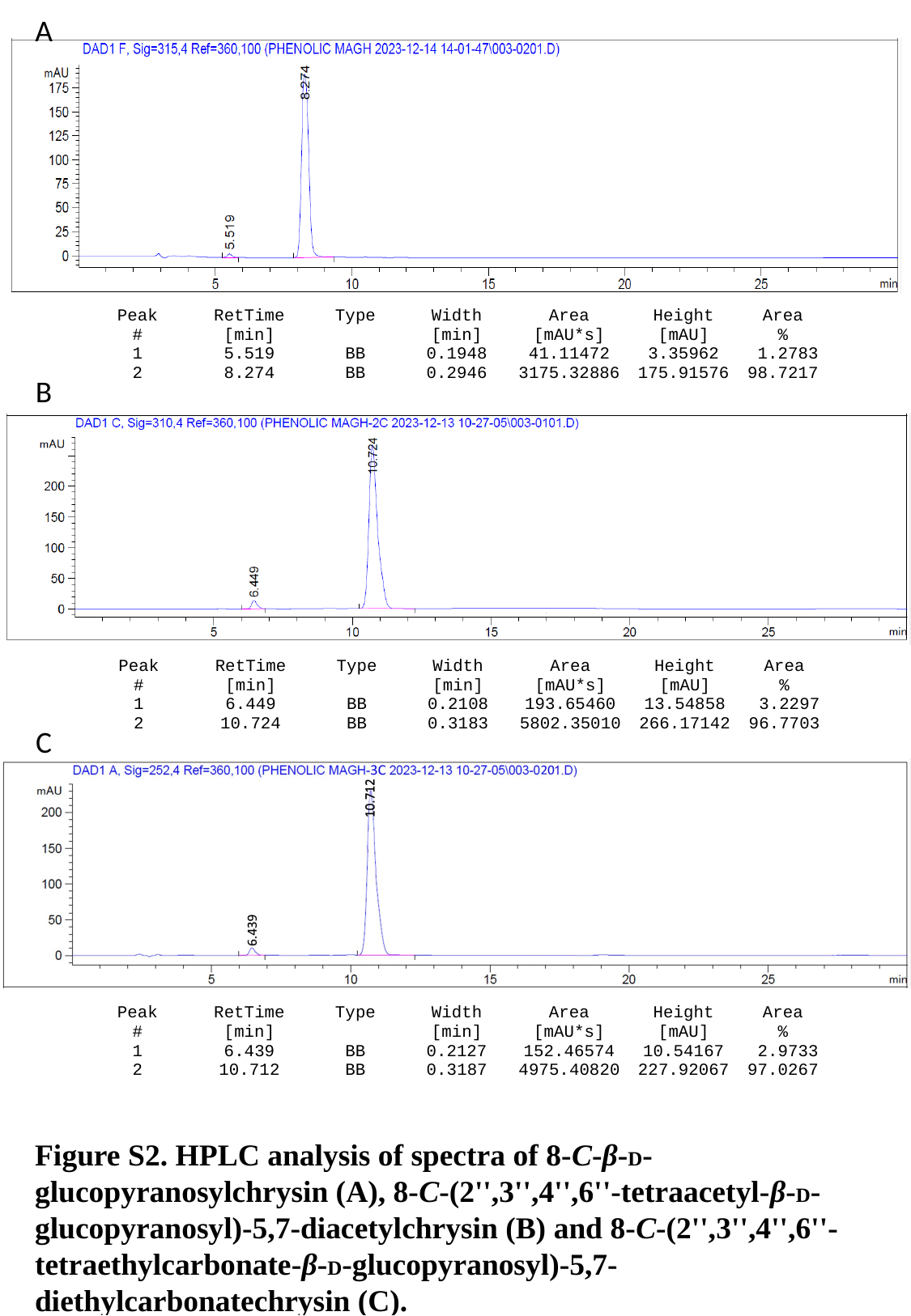

A
B
C
Figure S2. HPLC analysis of spectra of 8-C-β-D-glucopyranosylchrysin (A), 8-C-(2'',3'',4'',6''-tetraacetyl-β-D-glucopyranosyl)-5,7-diacetylchrysin (B) and 8-C-(2'',3'',4'',6''-tetraethylcarbonate-β-D-glucopyranosyl)-5,7-diethylcarbonatechrysin (C).

## Slide 4
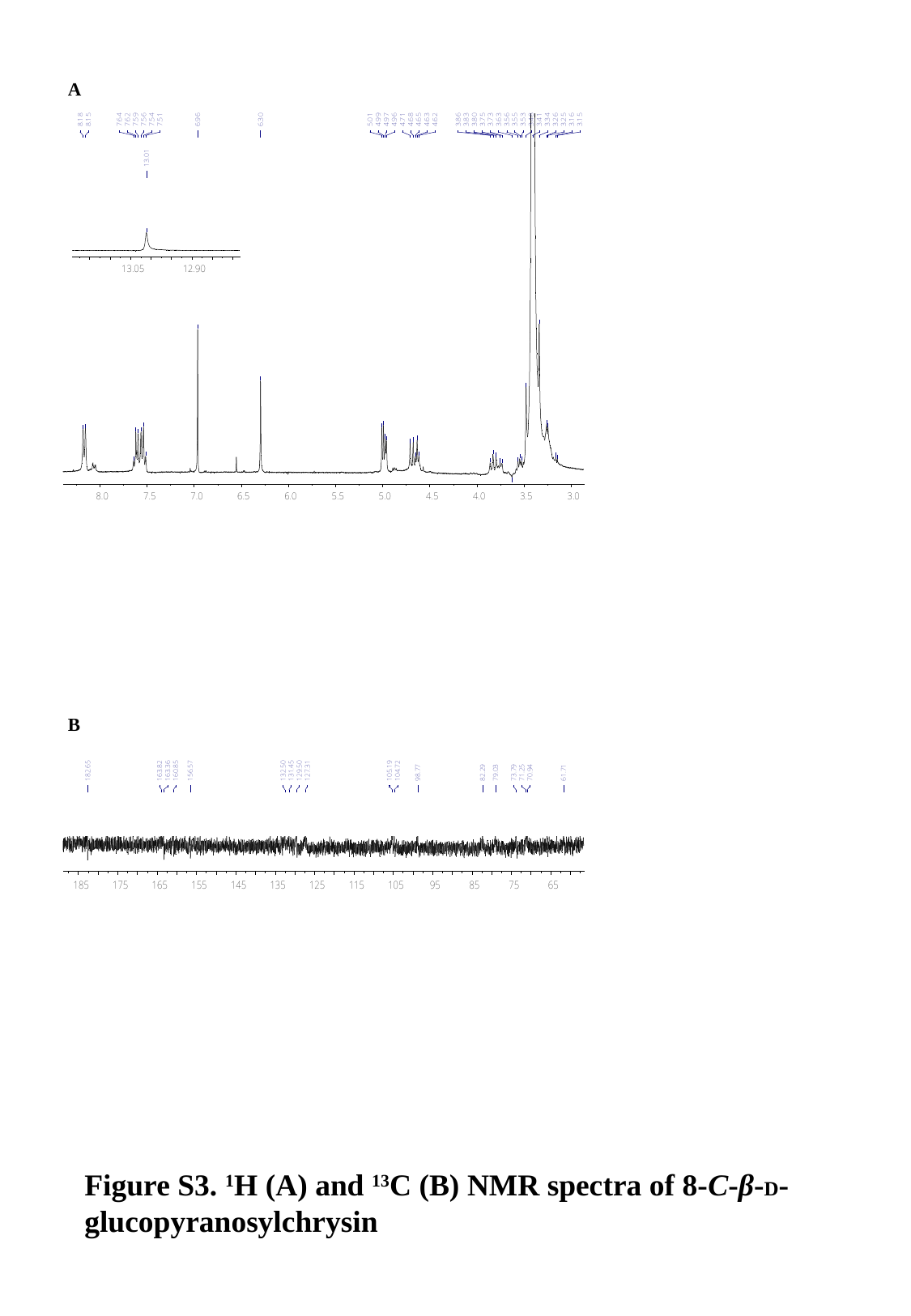

A
B
Figure S3. 1H (A) and 13C (B) NMR spectra of 8-C-β-D-glucopyranosylchrysin

## Slide 5
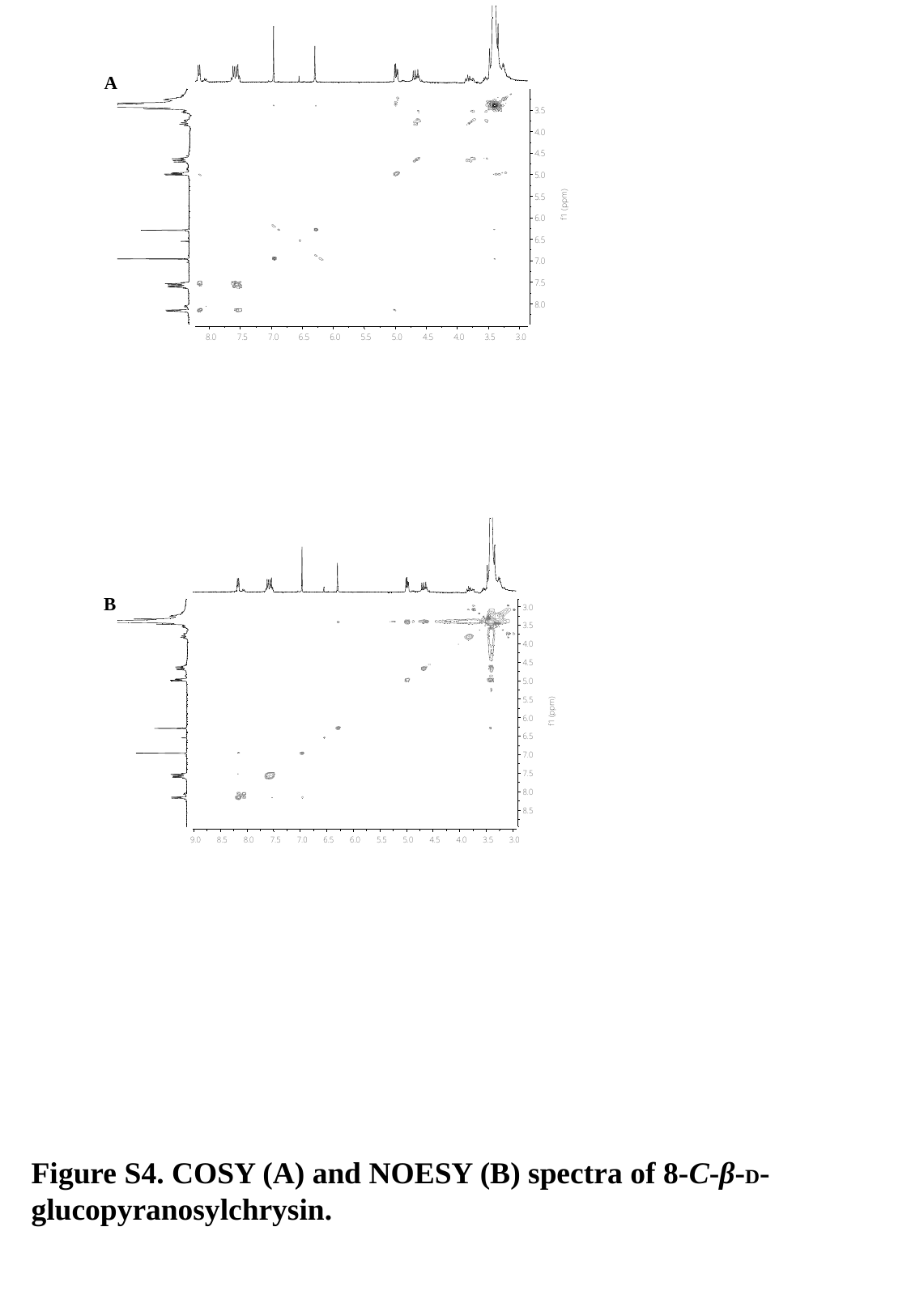

A
B
Figure S4. COSY (A) and NOESY (B) spectra of 8-C-β-D-glucopyranosylchrysin.

## Slide 6
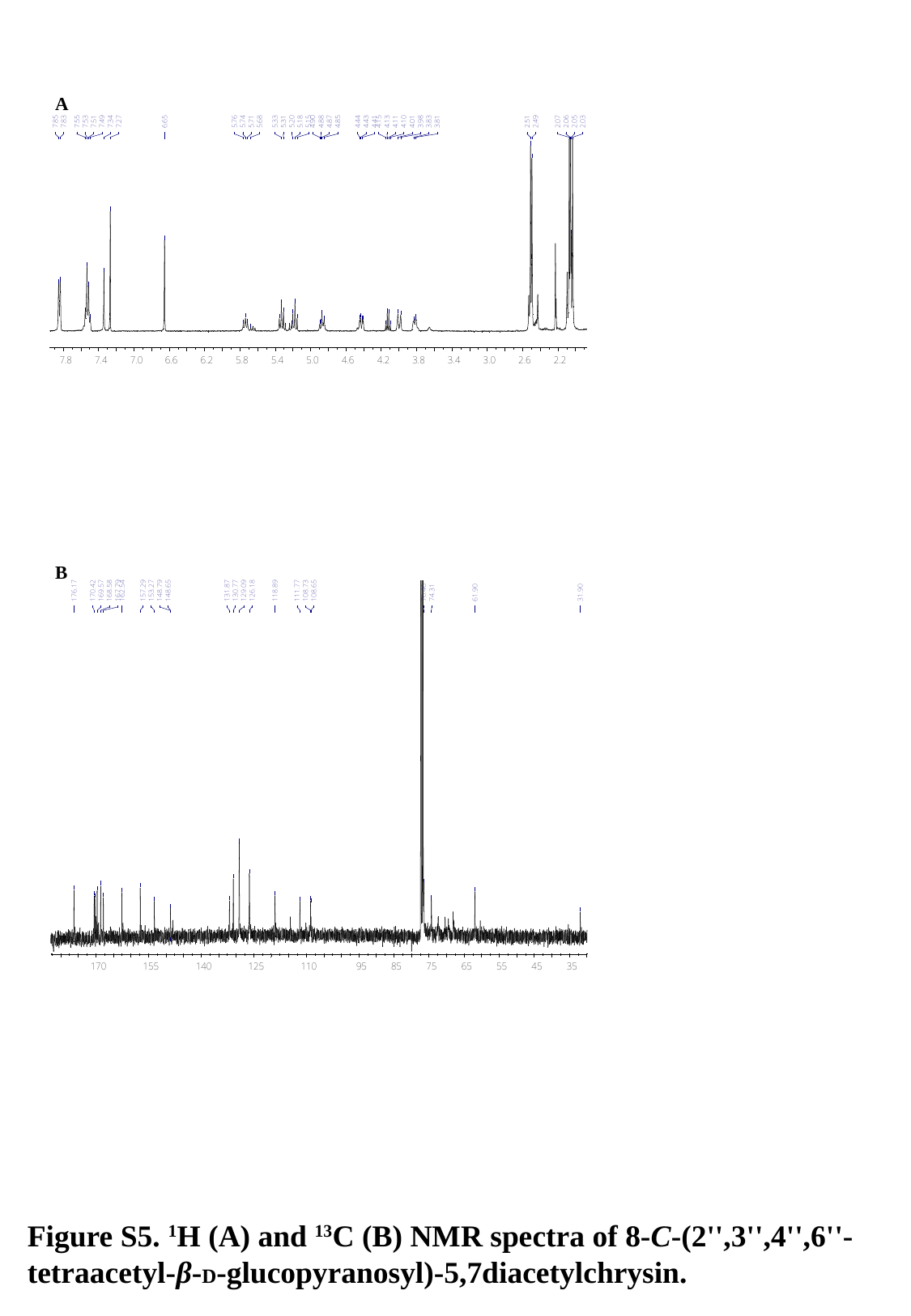

A
B
Figure S5. 1H (A) and 13C (B) NMR spectra of 8-C-(2'',3'',4'',6''-tetraacetyl-β-D-glucopyranosyl)-5,7diacetylchrysin.

## Slide 7
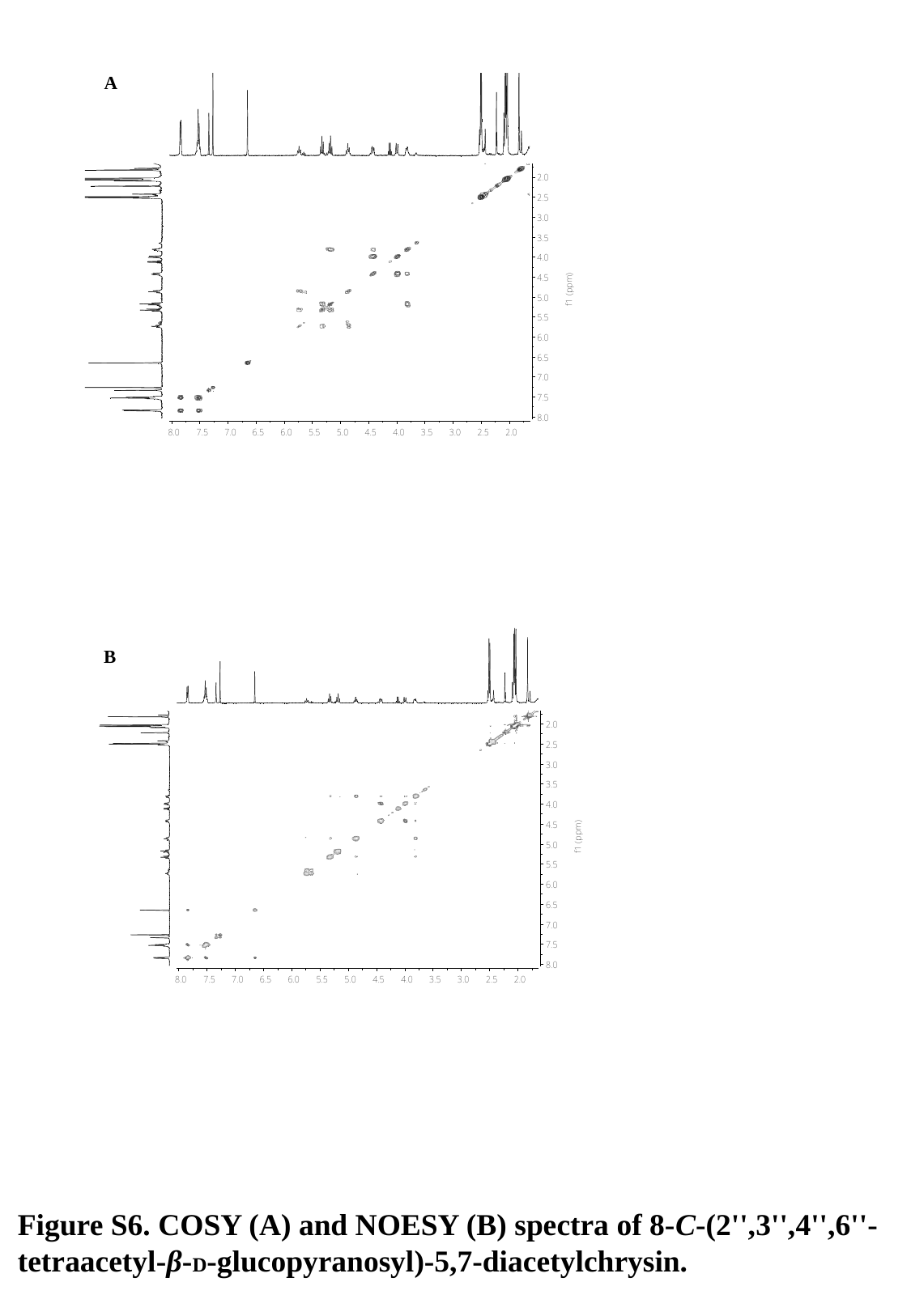

A
B
Figure S6. COSY (A) and NOESY (B) spectra of 8-C-(2'',3'',4'',6''-tetraacetyl-β-D-glucopyranosyl)-5,7-diacetylchrysin.

## Slide 8
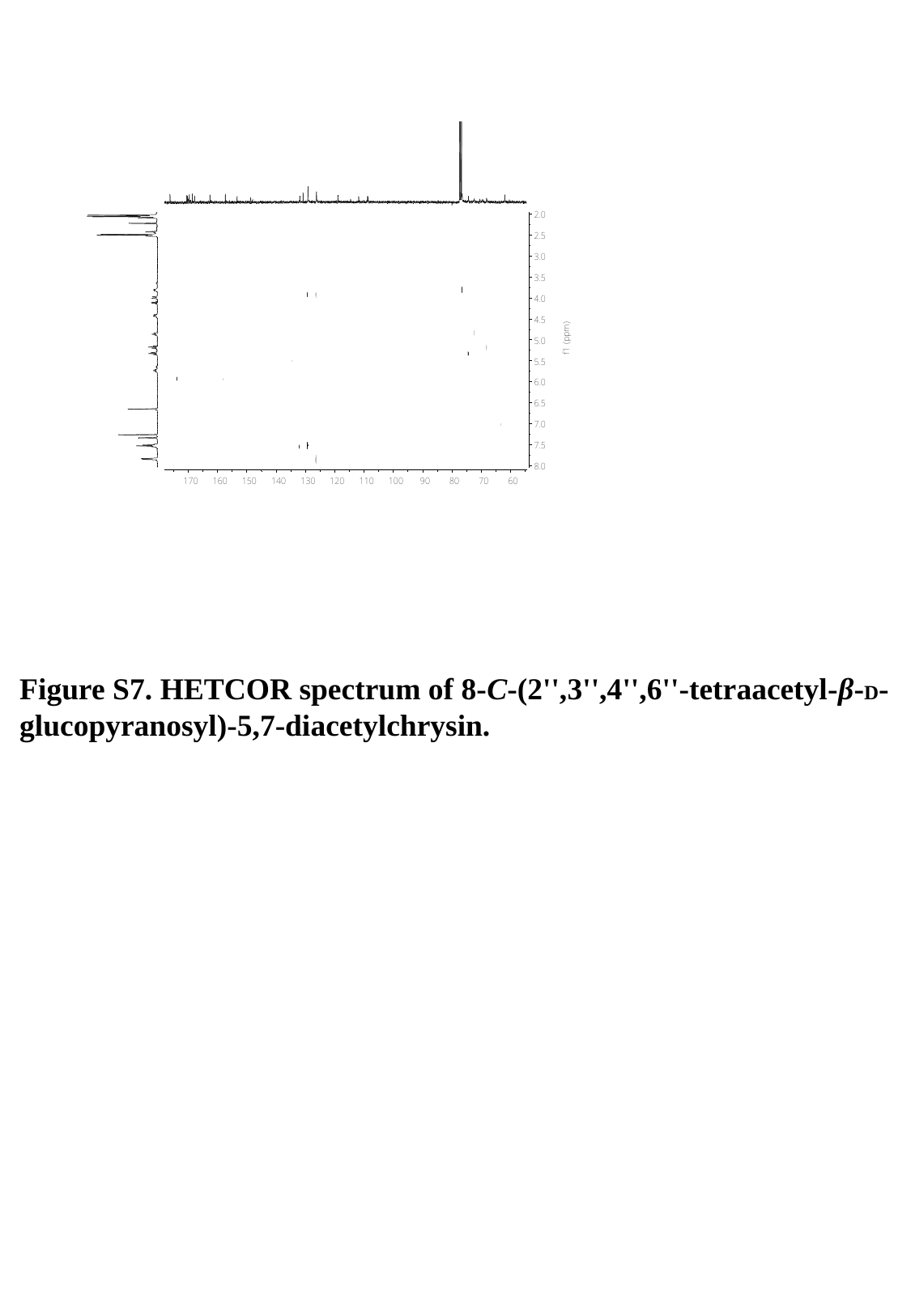

Figure S7. HETCOR spectrum of 8-C-(2'',3'',4'',6''-tetraacetyl-β-D-glucopyranosyl)-5,7-diacetylchrysin.

## Slide 9
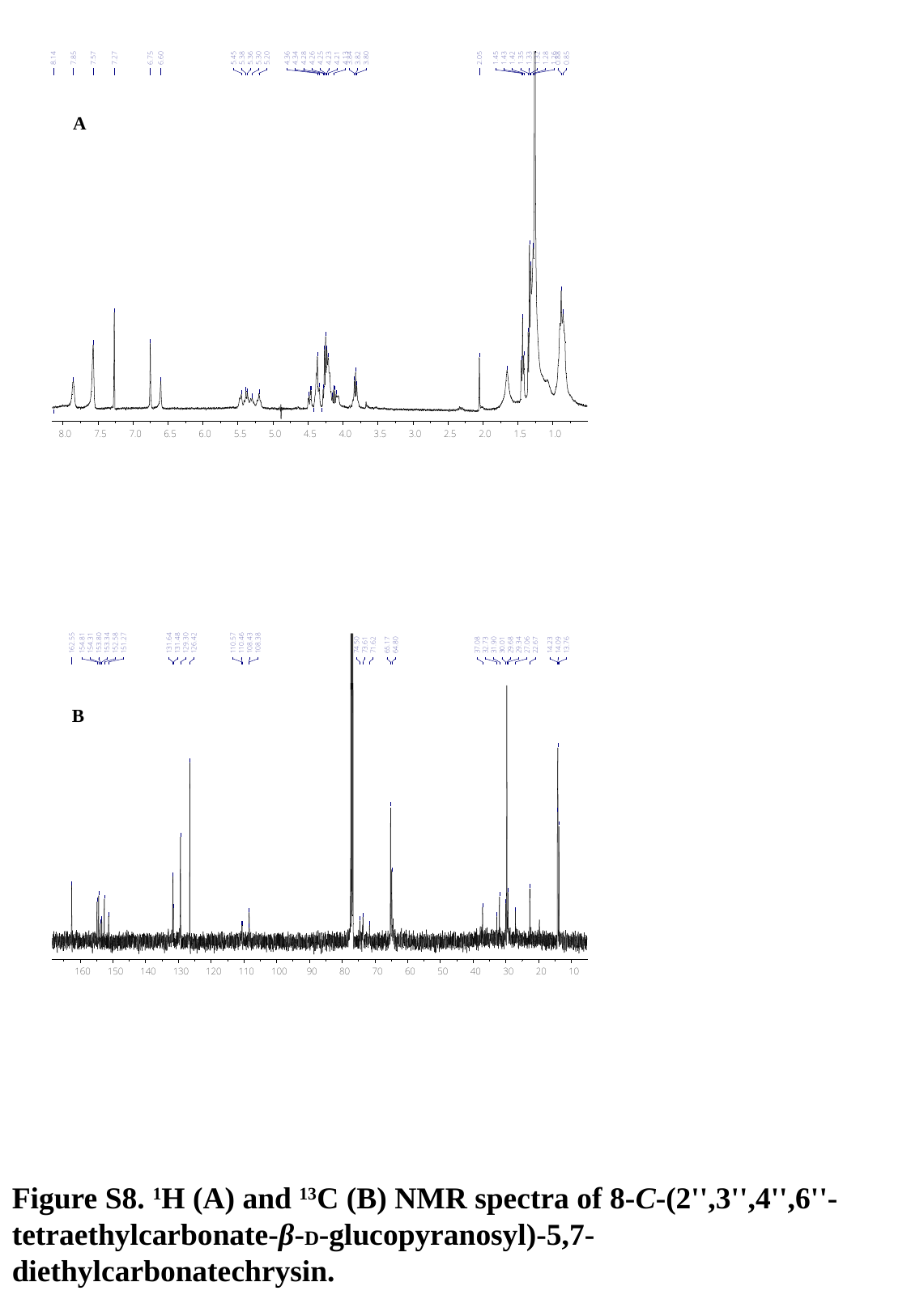

A
B
Figure S8. 1H (A) and 13C (B) NMR spectra of 8-C-(2'',3'',4'',6''-tetraethylcarbonate-β-D-glucopyranosyl)-5,7-diethylcarbonatechrysin.

## Slide 10
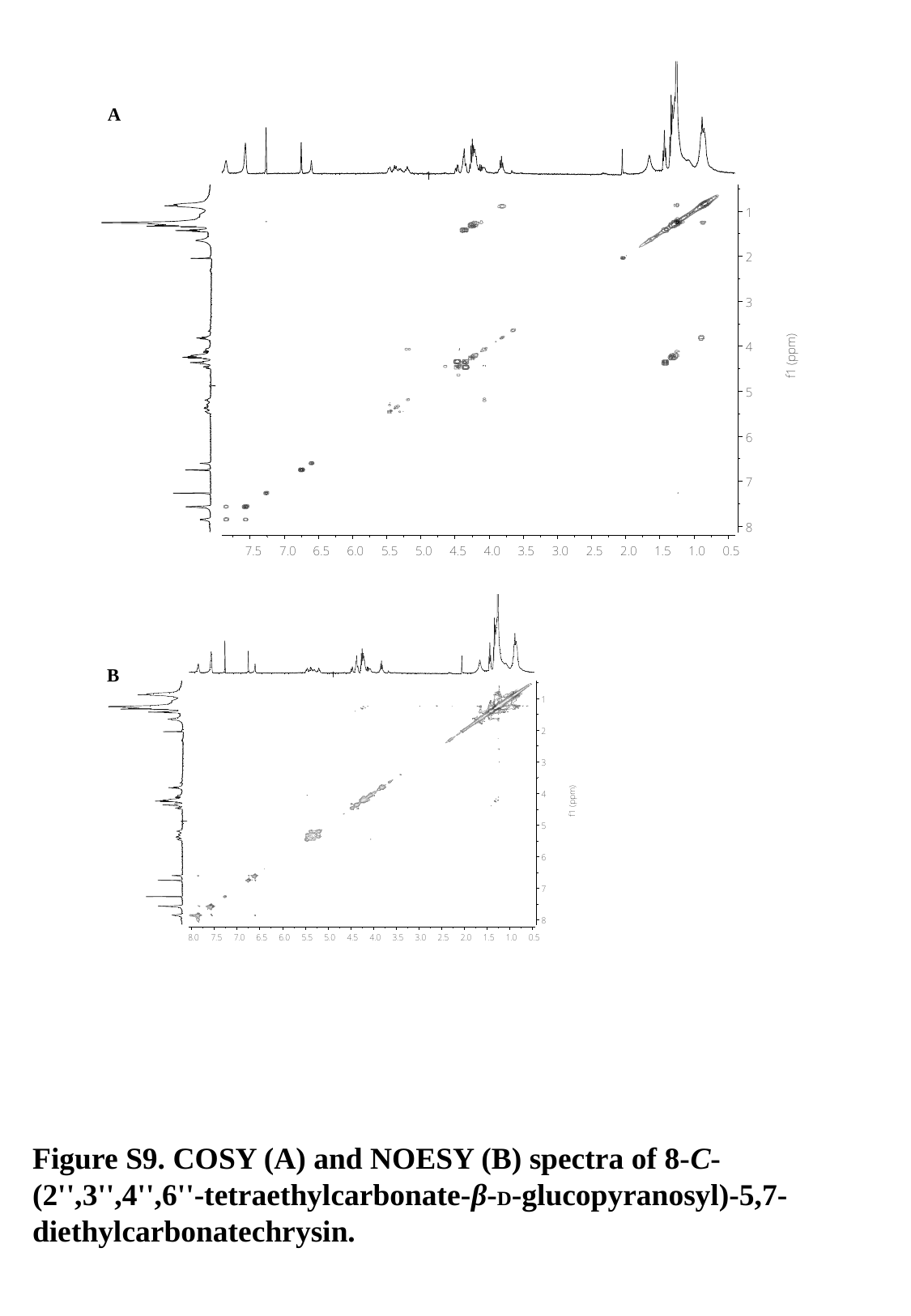

A
B
Figure S9. COSY (A) and NOESY (B) spectra of 8-C-(2'',3'',4'',6''-tetraethylcarbonate-β-D-glucopyranosyl)-5,7-diethylcarbonatechrysin.

## Slide 11
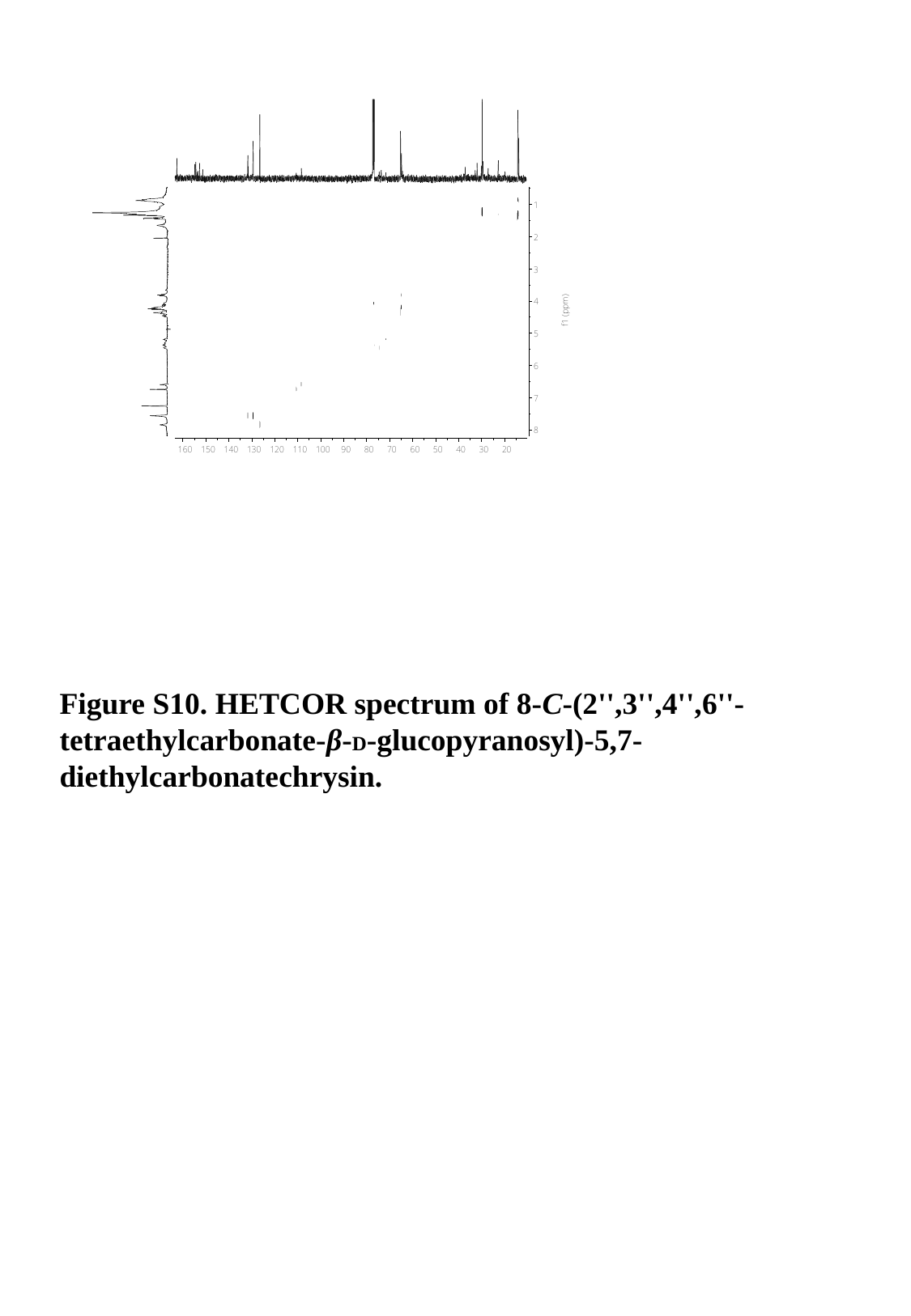

Figure S10. HETCOR spectrum of 8-C-(2'',3'',4'',6''-tetraethylcarbonate-β-D-glucopyranosyl)-5,7-diethylcarbonatechrysin.

## Slide 12
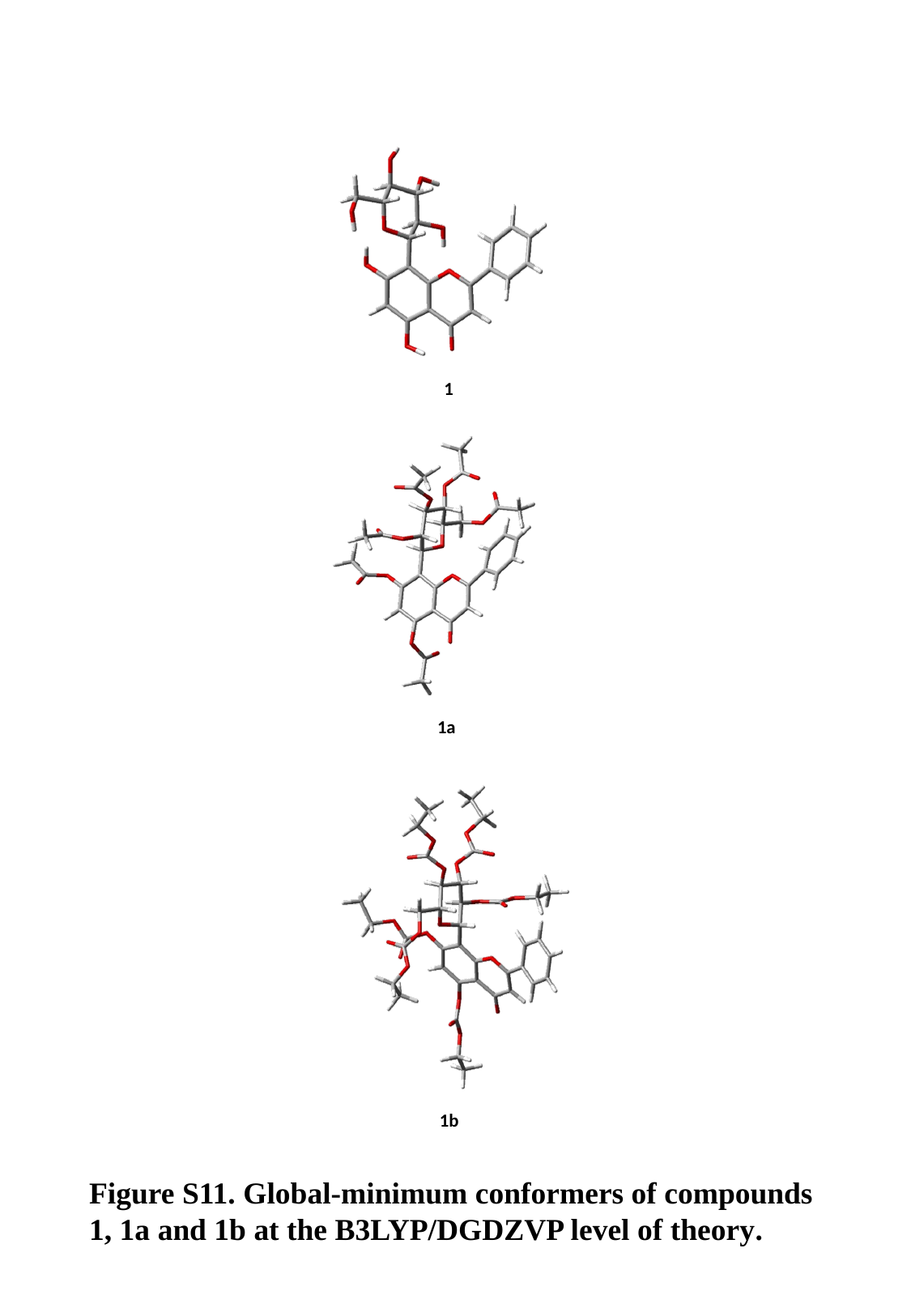

Figure S11. Global-minimum conformers of compounds 1, 1a and 1b at the B3LYP/DGDZVP level of theory.

## Slide 13
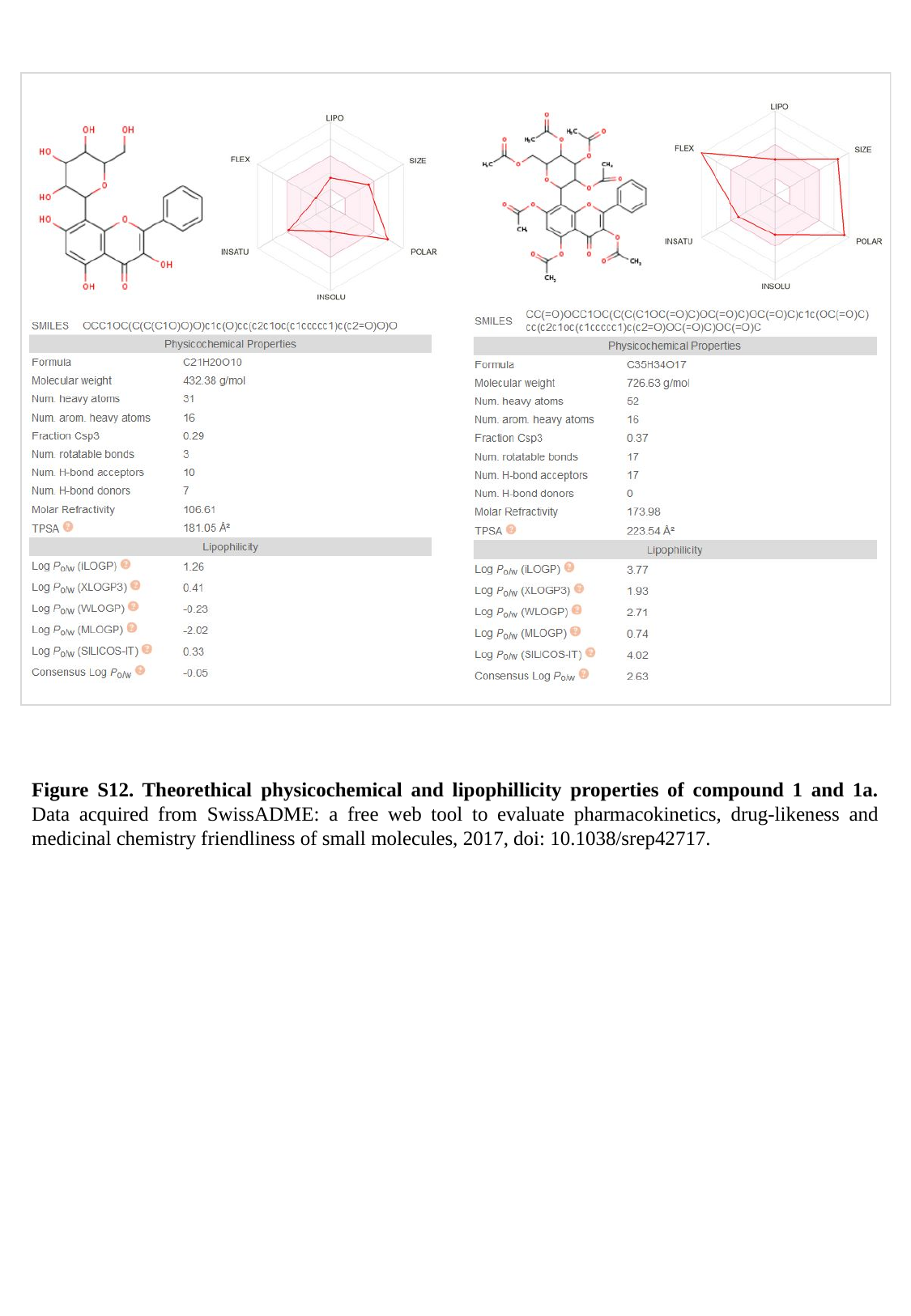

Figure S12. Theorethical physicochemical and lipophillicity properties of compound 1 and 1a. Data acquired from SwissADME: a free web tool to evaluate pharmacokinetics, drug-likeness and medicinal chemistry friendliness of small molecules, 2017, doi: 10.1038/srep42717.
